# Exploring Senior Phase teachers’ competencies in supporting learners with specific learning difficulties: Implications for inclusive education

**DOI:** 10.4102/ajod.v11i0.901

**Published:** 2022-08-31

**Authors:** Mubi F. Mavuso

**Affiliations:** 1 Department of Psychology of Education, School of Educational Studies, University of South Africa, Pretoria, South Africa

**Keywords:** learning support, specific learning difficulties, teacher competencies, inclusive education, barriers to learning

## Abstract

**Background:**

Teaching learners with specific learning difficulties requires competent teachers who can provide learning support. Competencies such as identifying learning difficulties, assessing learners, designing interventions such as curriculum differentiation and facilitating referral systems are crucial. However, Senior Phase teachers in South Africa seem to be challenged when it comes to providing learning support. Consequently, learners do not meet the desired learning outcomes.

**Objectives:**

The purpose of this study was to explore Senior Phase teachers’ competencies in supporting learners with specific learning difficulties in four mainstream schools.

**Methods:**

A qualitative research approach and phenomenological research design were used. Eighteen teachers who were members of the school-based support teams, including learning support educators, were selected through purposive sampling. Data were collected through individual and focus group interviews, the analysis of support forms and field notes. A thematic data analysis was used to generate findings.

**Results:**

The thematic data analysis revealed discrepancies relating to participants’ competencies in identifying language difficulties, short-term memory problems and contextual barriers. Also, participants differed in collaborating with peers, social workers, and the district-based support teams. Furthermore, some participants were able to design intervention programmes and facilitate internal and external referral processes.

**Conclusion:**

The study concludes that teachers have different competencies in providing learning support. Therefore, the Department of Basic Education should provide a clear practical learning support strategy in the Senior Phase mainstream schools as well as continuous professional development for teachers couple with monitoring.

**Contribution:**

It is envisioned that the study will contribute to understanding teachers’ competences in providing learning support for learners with specific learning difficulties in the senior phase. The study advocates for collaborative continuous professional teacher development focusing on interventions programmes to support learners with specific learning difficulties in the mainstream schools.

## Introduction

The purpose of the study on which this article is based was to explore Senior Phase teachers’ competencies in supporting learners with specific learning difficulties (SpLDs) in mainstream schools. Specific learning difficulties are common in schools. They include dyslexia, dyspraxia, dysgraphia and dyscalculia (Rowlands et al. 2013). They can also include visual processing, auditory processing, time management and sensory difficulties (Pumfrey and Reason 2013). Furthermore, SpLDs highlight the discrepancy between academic achievement and intellectual ability (Gresham and Vellutino 2010). Specific learning difficulties may be less readily identifiable, do not always have a clear physical basis and are more subject to different social contexts (Donald, Lazarus & Lolwana 2006). Therefore, the description of the concepts SpLDs is complex and constitutes various views from researchers (Donald, Lazarus & Lolwana 2010).

Teaching learners with SpLDs requires competent teachers who display positive attitudes towards learners (Sagor & Cox 2013). These attributes are critical to ensure that learners with SpLDs learn optimally and achieve the desired learning outcomes – more so because most learners who experience SpLDs have learning difficulties that are accommodated in mainstream schools, where they learn with their peers as part of a policy of inclusive education (Department of Basic Education [DBE] 2010a; Ferguson 2008).

Although such competencies are crucial, Senior Phase teachers in the mainstream schools seem to be challenged in providing learning support for learners presenting with SpLDs. This results in an alarming number of referrals to the Inclusion and Special Schools (ISS) unit that mediates learning support processes within an inclusive education context at the district office.

In the current study, learners who presented with SpLDs were between the ages of 14 and 18 years. They could not read or write in their African languages or in English as a first additional language. Some could not recognise or write their own names; they performed poorly and had repeated grades several times. Their referral to the ISS unit that mediates the learning support process within the inclusive education context occurred when they were already in Grade 7 and Grade 9. The observation suggested that teachers lacked learning support competencies such as identifying learning difficulties and assessing learners. They could not design and implement interventions such as curriculum differentiation. Furthermore, they struggled to facilitate accommodations and referral systems.

Lack of such competencies happened despite availability of education policies which are aimed at promoting inclusive education and, by implication, providing learning support. For instance, the Education White Paper 6 (EWP6): Special Needs Education – Building an Inclusive Education and Training System (Department of Education [DoE] 2001) was intended to promote equal and quality education. The Guidelines for Full-Service Inclusive Schools (DBE 2010b) acknowledge that certain learners require individualised attention and suggest that these needs could be determined through the strategy on screening, identification, assessment and support (SIAS) process that utilises Support Needs Assessments (DoE 2008, 2014). Therefore, the implication is that teachers should be competent in providing learning support.

On the one hand, scholars such as Woolfson, Grant and Campbell (2007) state that mainstream teachers have not always responded positively to the inclusion of learners with learning difficulties. Furthermore, Dreyer, Engelbrecht and Swart (2012) point out that teachers in the mainstream schools have not been adequately equipped to deal with barriers in diverse classrooms. Teachers were prepared to teach in either special schools or mainstream schools (Donohue & Bornman 2014). In practice, this means that those who teach in the mainstream schools were not exposed to supporting learners with SpLDs and their learning needs.

Despite these observations, little is known about the competencies of teachers in providing learning support in the Senior Phase mainstream schools and how their competencies influence learning support processes. Wentzel (2016) investigated learning support for children with mild intellectual difficulties in primary schools in Port Elizabeth within the Eastern Cape province and found that untrained teachers struggled to support learners, whilst Chataika, Kamchedzera and Semphere (2017) found that mainstream primary school teachers in the Lilongwe urban district of Malawi were challenged in planning instructional strategies for inclusive classrooms.

Considering the proclaimed inadequate preparedness of mainstream teachers for inclusive education practices in supporting learners with SpLDs, it was deemed necessary to explore the competencies of teachers, as they could influence learning support processes.

The research questions were phrased as follows:

- What are the teachers’ competencies in identifying SpLDs?
- What are the teachers’ competencies in implementing learning support processes?
- What are the teachers’ competencies in collaborating?
- What learning support interventions do teachers design?

### Teacher competencies in providing learning support

Teachers are better positioned to provide learning support in inclusive classrooms (De Jager 2013; Forlin & Chambers 2011). Thus, teachers require multiple competencies, such as knowledge and skills related to teaching, in order to be able to respond to diverse learning needs (Chireshe 2013; Mavuso 2015; Nel, Nel & Hugo 2013a). Such competencies include identifying learning difficulties (DBE 2014; Zwane& Malale 2018); assessing learners (Kanje & Mthembu 2015; Venter 2012); differentiating the curriculum (DBE 2010; Dednam 2011; Lake 2010); using different instructional strategies (Donohue & Bornman 2014; Lake 2010); referral systems; implementing accommodations such as large print; amanuensis or use of a scribe; additional time; using assistive devices such as computers and Braille; an interpreter for deaf learners, rephrasing for deaf learners and use of a separate venue (DBE 2010, 2011; Venter 2012); and facilitating internal and external referrals and collaboration (DBE 2014; DoE 2001). Importantly so, learning support is part of teaching and learning and should not be regarded as a separate process.

Identifying learning difficulties is an ongoing process. Therefore, identification means that teachers observe learners as they teach and record their observations on what the learners can do; they need to note areas that pose barriers to learning. These observations should be communicated to parents, who could assist with additional information for screening (eds. Aro & Ahonen 2011). The competencies required for this mean that teachers must be knowledgeable about what factors constitute SpLDs (DBE 2014).

Linked to observations, teachers should be able to screen learners for academic performance and not necessarily for diagnosing a learning disability (Farrall, Wright & Wright 2015). This is because a diagnosis requires specialist competencies and training. For this reason, Venter (2012) suggests that teachers should consult specialists in the community to obtain more information about other barriers and disabilities. The provision made by the DBE in South Africa is that teachers should work collaboratively with the school-based support teams (SBSTs) and the district-based support teams (DBSTs) (DBE 2014). As a result, educational psychologists within the DBSTs should be contacted to assist with specific identification, assessment and support. Such a practice is recommended as not every school has private educational psychologists that could assist teachers within their schools. However, parents should not be excluded from the process, as they can provide valuable information regarding their observations about the learner.

Teachers’ competencies in assessing learners involve collecting, interpreting, documenting and using information about a learner (Kanje & Mthembu 2015; Lombard 2010; Venter 2012). Therefore, assessment should be used as an integral part of teaching and learning and should be viewed as continuous and not as a once-off activity (Landsburg 2011; Looney 2011). Researchers such as Herman, Osmundson and Silver (2010); Grigorenko (2009); and Vogel (2011) argue that assessment informs teachers about the learner’s progress regarding meeting learning outcomes. Thus, it can be used by teachers to improve their teaching, identify the strengths and learning barriers encountered by the learners and inform parents about the progress of the learner.

In addition, teachers require competency in using assessment to inform learners regarding what is expected from them, how to improve their learning and what skills and knowledge they require to progress in their learning (DoE 2011). Consequently, different forms of assessment can be carried out to enhance learning. For instance, to establish prior learning, teachers should be able to use a curriculum-based diagnostic assessment. The observations from such an assessment could be used to plan and design individualised instruction as a form of support (DBE 2014). Walton (2012) states that individualised support plans are necessary and can benefit learners who require structured and individualised interventions and support.

In assessing learners, teachers should also be competent in using formative assessment as a holistic approach to teaching. Formative assessment is necessary as part of the continuous identification of gaps in learning; it is mostly beneficial for learners with learning difficulties, as it minimises inequality amongst learners (Kanje & Mthembu 2015; Looney 2011). Hence, as an intervention strategy, formative assessment should be carefully planned and aligned with what should be learnt. It should provide for the learner’s unique learning needs to ensure that the learner progresses.

Related to formative assessment is the competency of giving learners constructive feedback. Such an action recognises that learners are not spectators in their learning; they can also construct their learning. For this reason, researchers such as Yong and Carless (2013) contend that learners have preferences when it comes to feedback from teachers, and they consider it to be beneficial. Learners can also benefit from feedback as they become aware of what is expected of them. The advantage of giving feedback is that it can help teachers to see progress made by learners and to prepare further scaffolding for learners. In essence, continuous learning support could be evident.

Similarly, curriculum differentiation forms part of key competencies for ensuring the successful provision of learning support (DBE 2010a; Dednam 2011; Lake 2010). Differentiating the curriculum means that teachers are competent in establishing learners’ readiness to learn, acknowledging their differences and in planning and implementing appropriate instructional methods (Venter 2012).

Differentiation also includes adjusting the content and ensuring that different methods are used to impart the information and skills required by learners to learn (Merga 2020). It also involves the process of planning the learning activities in a way that engages learners and relates to the product that shows evidence of applied skills and knowledge of what has been learnt (Bornman & Rose 2010). Researchers such as Nel et al. (2013a) contend that curriculum differentiation includes scaffolding. For this reason, Woolfolk (2010) speaks of systematic scaffolding and indicates that for it to be beneficial, it should be varied for individual learners. The researcher proposes that teachers should be flexible when implementing scaffolding as a learning support strategy.

Differentiations could also be used by adapting teaching strategies (Donohue & Bornman 2014; Lake 2010). Dednam (2011) advances that adapting teaching strategies involves cognitive support that equips learners with skills to actively attend and perceive stimuli through using auditory and visual senses and building learning experiences through self-activity. During the process of differentiation, learners are advised to use those strengths that best help them to learn, and they should be taught different study techniques that will assist them to memorise information.

Teachers should also be competent in facilitating accommodations as a strategy for accessing the curriculum (DBE 2010a; Miller 2009; Nel et al. 2013b; Venter 2012). Hence, teachers are viewed as mechanisms that create pathways to learning without changing the learning outcomes. Provisions made for accommodations include using scribes, large print, additional time and writing in a separate venue (DBE 2010).

Importantly, teachers could enhance learning support processes through adopting universal design for learning (UDL) principles by ensuring that learning material is presented using multiple formats of media that provide multiple pathways for students’ actions in accessing the information and using multiple ways to engage students’ interest and motivation (Browder et al. 2008, 2010; Walton 2012).

It is also important that teachers become competent in collaborating with other teachers, DBSTs and other professionals when a need arises to alleviate blockages that could inhibit learning support (DoE 2005, DBE 2014). Training of teachers on learning support competencies occurs through workshops conducted by the officials from the ISS unit in collaboration with other relevant stakeholders and through continuous teacher development.

## Study design

A qualitative research approach was used in the study by involving Senior Phase teachers from four mainstream schools. The approach was chosen because of its descriptive, explorative and explanatory nature (Merriam 2009). It could also assist in uncovering the meaning participants attach to the phenomenon of SpLDs and learning support. Thus, the experiences of Senior Phase teachers were described within their unique context and in detail to understand their beliefs (Babbie & Mouton 2008; Henning 2004).

The research design was phenomenological, for the purpose of understanding the meaning teachers attached to providing learning support for learners presenting with SpLDs in mainstream schools. It was used to understand participants’ perceptions, within their context (eds. De Vos at al. 2006). It assisted in interpreting the meaning the participants gave to their everyday lives and experiences (Creswell 2007), and it offered a descriptive, reflective and engaged mode of enquiry (McMillan & Schumacher 2010).

### Selection of participants

A purposive sampling was used in this study. It included Senior Phase teachers (grades 7–9) who were members of the SBSTs and were therefore allocated roles of providing learning support. Specifically, they included subject teachers, heads of departments in Life Orientation and learning support educators who were invited to participate on a voluntary basis. Participants had also interacted with learners who presented with SpLDs.

They were selected because they were the holders of the data needed for the study (Creswell 2009) and could provide the richest data to allow the researcher to gain insight into how learners presenting with SpLDs were supported (Cohen, Manion & Morrison 2008). One would therefore expect that the participants were competent to provide learning support. It was necessary to understand how their competencies enhanced or hindered the learning support processes. The participants had varying levels of teaching experience. After the purpose of the study was explained to them, the participants signed consent forms. They were assured of confidentiality and anonymity. The participants were from four schools, three of which were in a township and one was in the city centre. The schools were selected because they had a substantial number of referrals of learners who presented with SpLDs.

### Data collection methods

Data were collected through semistructured individual and focus group interviews, the analysis of support records and a reflective journal. The interviews were conducted in four schools considered to be convenient for all participants. The interviews lasted for approximately 45 min – 60 min. There was no interference with teaching and learning.

The semistructured, open-ended questions for both individual and focus group interviews focused on how teachers provided learning support for learners presenting with SpLDs. The questions were phrased to elicit competencies identifying learners with SpLDs’ difficulties, interventions they used and the learning support processes they used. The questions were asked in a flexible manner. The interviews allowed the participants to reflect their reality and helped the researcher to obtain answers to the research question (Babbie & Mouton 2008; De Vos et al. 2011). Six participants were interviewed individually. They consisted of four teachers and two learning support educators.

Two sets of focus group interviews were conducted with participants who shared similar experiences regarding the topic being investigated (Babbie et al. 2008; Greeff 2009). Each group had four teachers from each school who did not participate in the individual interviews and two learning support teachers. They allowed space for participants to get together and create meaning amongst themselves, rather than doing individually, thus giving them an opportunity for shaping and reshaping opinions.

Support forms were used as documents for corroborating the data from the interviews and to enhance the trustworthiness of the study (McMillan & Schumacher 2010). These documents are used by teachers to document their observations and the interventions they use. They excluded confidential medical and psychological documents, as it was not the intention of the study to focus on such records. As De Vos et al. (eds. 2009) advise, field notes were included to record what the researcher heard, saw, experienced and thought during the process of interviewing.

### Data analysis

Data analysis was conducted in the form of thematic content analysis (Henning, Gravett & Van Rensberg 2004). The process included transcribing the interviews, reading each transcript several times to get a sense of the data, breaking down the data into manageable sections, identifying differences, similarities, relations and interactions within themes, assigning codes through labelling each section of the data related to the research question (Creswell 2009), testing the emergent understandings and representing and visualising the findings (eds. De Vos et al. 2009). Meanwhile, support forms were interpreted by ascertaining how teachers documented the learning support processed.

### Trustworthiness of the study

Trustworthiness was ensured through credibility, which was achieved through prolonged engagement in the field until data were saturated; persistent observation by looking at what was happening in the field as the author entered each site, as well as during interviews; peer debriefing by discussions with peers and presenting in the seminars and being critiqued; reflexivity by writing notes on the author’s thoughts after interviews; transferability, which was achieved through purposive sampling and a thick description of the findings; dependability by using multiple data sources; and confirmability by triangulation and audit trail (Lincoln & Guba 1985). Feedback was provided to all the participants, allowing them to corroborate the findings (Babbie & Mouton 2008; eds. De Voset al. 2006).

### Ethical considerations

Ethical procedures included obtaining an ethical clearance certificate from the university where the study was conducted (reference number 2013074) and obtaining permission to conduct the study from the Gauteng Department of Education, the district director and school principals. Ethical clearance to conduct the study was obtained from the Faculty of Education Research Ethics Committee, University of Johannesburg. Consent was also given by the participants. Other ethical measures included confidentiality, anonymity, respect, giving feedback to the participants and ensuring that the participants were not harmed.

## Findings and discussions

The findings revealed that teachers had different competencies when it came to the provision of learning support. Their competencies differed in terms of how to identify SpLDs, designing and implementing interventions, processing internal referrals and collaborating with external stakeholders. These are discussed in the following section.

### Teachers’ competencies in identifying specific learning difficulties

The findings indicate discrepancies amongst members of the SBST on how to identify learners presenting with SpLDs, with some members displaying competency whilst others lacked competencies. The participants who were able to identify these learners could articulate their observations by mentioning symptoms such as significant language difficulties, difficulties with reading and writing, difficulties with spelling and short-term memory problems, and they referred to contextual barriers that contribute to SpLDs. By contrast, those who seemed unable to identify learning barriers mentioned that they could not describe the SpLDs. They knew that something was wrong, but they could not articulate it.

The following are selected extracts of what was expressed:

‘As a teacher, you can immediately identify a learner that has a problem in English. A learner will look at a word for more than a minute, even if you give time to read. Some would skip words when they read. Their vocabulary in English is poor and not at the Grade 7 level.’ (Participant 4, female, learning support educator)

‘So you [*the teacher*] can easily see learners with communication problems. You observe that they struggle to communicate because of language difficulties. So learners with language difficulties cannot give you answers when you ask them a question. I have seen this when I speak in English.’ (Participant 3, female, HOD/SBST coordinator)

Contrasting views were expressed as follows:

‘You are not sure what you see. You see that something is not OK … [*pause*] but are not sure. So for the sake of time, you continue teaching your subject.’ (Participant 14, female, grade 8 and 9 teacher)

‘You recognise the problem of the child when it is towards the end of the year, when it is already late or when the child would have been failing by then.’ (Participant 17, male, grade 8 and 9 teacher)

Similarly, Zwane and Malale (2018) found that high school teachers in Swaziland felt incompetent in identifying learners with learning challenges. Therefore, identifying learners presenting with SpLDs is of paramount importance. It can assist in preparing appropriate interventions for supporting the learner (DBE 2014). Differing competencies amongst teachers is therefore problematic, as it can delay referral processes for learners. Thus, the findings have a bearing on the continuous professional development of teachers to strengthen their competencies and to promote collaboration amongst teachers.

Linked to competencies in supporting language difficulties, participants also expressed their experiences in identifying reading and writing difficulties as follows:

‘I could clearly see that they don’t know how to write, because I don’t think they had proper foundation at the primary level. Their spelling is poor and not at grade level. If you give them a paragraph to write, you identify incomplete words and sentences.’ (Participant 6, female, grade 7 teacher)

‘Once a learner glances at the book whilst reading, I start to think that there could be problems. Others add words which are not in the text they are reading. Those who struggle with writing, you just cannot read their handwriting.’ (Participant 18, male, grade 8 and 9 teacher)

In contrast, the following views were reported:

‘We should be trained how to identify different learning difficulties. This is important, especially for those learners who cannot read and write. Most of us are clueless about reading and writing problems.’ (Participant 8, female, learning support educator)

‘I continue to teach what I have to teach, even if I see that something is wrong. I cannot tell what is wrong. Therefore, I focus on the lesson to complete the syllabus. So I also think we should be trained intensively on how to support learners.’ (Participant 16, female, grade 8 and 9 teacher)

As Pretorius et al. (2016) indicate, training of teachers on reading is essential as it might be linked to lack of competencies in teaching reading. Supporting learners with reading difficulties serves as a basis for offering learning support, because the inability to read affects understanding and adversely influences learning. Failure to identify this prevents learners from benefiting from alternative methods of learning support. It may therefore be necessary to re-examine the structure and functions of the SBST, the manner in which training is done and how it functions.

### Competencies in implementing learning support processes

This subtheme highlighted competencies relating to learning support processes. Such competencies include practical steps that teachers should take in providing learning support, which, in addition to identification, include developing individual support programmes, using an internal and external referral system and collaboration.

Selected views were as follows:

‘Because there are different learning barriers, when it comes to academic fields, you do adaptations; for example, time is for learners who are slow in class; you give them a little extra time. Others, for example with visual problem, you make the writing bigger.’ (Participant 10, female, HOD/SBST coordinator)

‘I try to translate, give the learner examples, the similarities and the opposite of the words [*antonyms*], you know, for the learner to understand what the vocabulary is.’ (Participant 5, female, learning support educator)

However, conflicting views were expressed as follows:

‘Because I teach many subjects, I do not know how to plan a programme to support learners. I may try, but I do not know how to do that in all subjects.’ (Participant 3, female, HOD/SBST coordinator)

‘I think the learning support educators must support educators in class with their adaptations, because educators don’t know how to. I also do not know what to do if a learner can’t read or write.’ (Participant 13, female, grade 8 and 9 teacher)

‘It is my second year of teaching. I am surprised to see many children with many learning problems. Even if I want to assist, I have no clue where to start in planning and writing support programme. What is also surprising is that I did not learn such at the university.’ (Participant 2, female, grade 7 teacher)

Dalton, Mckenzie and Kahonde. (2012) also reported that planning and working collaboratively by teachers and therapists benefited learners. Therefore, one could gather that the participants had varying competencies in classroom practices and implementing processes, as envisioned by the DBE. Such views also showed that the systematic planning of learning support processes could be hampered by work overload and blurred expectations of the roles of teachers in relation to the provision of learning support. The subtheme also highlights the problem of preservice training, where inclusive education is an option in teacher training as an elective and not infused in different modules.

### Competencies in collaborating

The study also revealed that to some extent, teachers do work collaboratively but that it is contextual and varies from school to school and from participant to participant. In the same school, for instance, some teachers worked collaboratively as peers as recommended by the DBE, whilst others chose to collaborate with social workers and the DBSPTs in the community. It also seemed that some teachers worked collaboratively in an informal manner.

The participants expressed themselves as follows:

‘One of our HODs [*heads of departments*] in school is actually qualified; she has a senior degree in learning support. So that’s one person that when you are stuck, you will literally go and ask her.’ (Participant 3, female, HOD/SBST coordinator)

‘You need someone for counselling before you can start with curriculum issues. So I refer learners to the local social worker. Some of the cases, I refer them to the district.’ (Participant 17, male, grade 8 and 9 teacher)

‘Teachers are not collaborative enough to be at ease to do case discussion. In my view, it is better to work with other people in the school and outside the school. People may have solutions to the problem.’ (Participant 18, male, grade 8 and 9 teacher)

However, others said:

‘With me, I have to rely on myself and do everything. I only teach and try to complete the syllabus. Who can really help me when everyone is concerned about their workload [*rhetorical*]?’ (Participant 13, female, grade 8 and 9 teacher)

‘To be honest, I do not know the role of the SBST, so what is the use? It is just important to teach and focus on all learners and not just one learner.’ (Participant 14, female, grade 8 and 9 teacher)

‘In my grade, we focus on the syllabus. I do my work. If one learner struggles, I just teach and focus on the rest of the class. In my heart, I know that what I am doing is not OK. I don’t think anyone can help me.’ (Participant 7, female, grade 7 teacher)

Collaboration amongst stakeholders within the education system has long been regarded as essential, but as Lerner and Kline (2006) assert, to be successful it must be based on certain principles, such as establishing a common goal and ensuring that it is voluntary and carried out by people who take responsibility for their actions. There should also be recognition of equality amongst partners and a sharing of accountability for the outcomes as well as the resources. Teachers need to be supported by both the general teacher and the special educator. These principles are also embedded in the policy guidelines for the establishment of SBSTs (DoE 2005).

### Analysis from support forms

It emerged that teachers could name few SpLDs, such as reading and writing difficulties. However, they did not elaborate on reading errors like omissions, substitutions and additions of words. Teachers considered calling parents and giving extra work as learning support interventions. Information about interventions such as curriculum differentiation, scaffolding, using individualised learning support programmes and using learner profiles to establish existing difficulties and support provided in previous grades was not captured. Additionally, the records indicated that parents were called but did not come to schools, without elaborating how were the parents called and how were such actions considered as support strategies. Support forms reflected that there were no strategies that worked. Furthermore, although the support forms required parents’ signatures, such signatures were missing in most support forms. In some cases, participants indicated that parents did not respond when they were invited even though the *South African Schools Act* (1996) and the EWP6 (DoE 2001) indicate that parental involvement is an important aspect of learning. The dates on the forms also suggested that teachers completed support forms when they were about to submit to the district or when placement for specific learners was requested by parents.

Monthly reports completed by Learning Support Educators reflected that they used specific terminology such as numeracy, literacy, SpLDs like possible dyslexia, a serious language barrier or significant language difficulties, spelling backlog, as well as emotional, socio-economic, systemic, individual and pedagogical factors as their evidence of providing learning support. The records also showed that they could elaborate on such terminology and how they collaborated with other stakeholders like parents, the district officials and social workers.

### Field notes

As a researcher, the author observed participants expressing their thoughts openly about how they provided learning support. Their thoughts assisted in extracting the competencies they had.

## Conclusion, implications and recommendations

This study has found that teachers have different competencies when it comes to providing learning support. Teachers are at the centre of facilitating learning support processes; hence, their competencies can enhance or hinder learning support. The problem is that most teachers have not been formally trained in inclusive education and specifically not in supporting learners presenting with SpLDs. This places pressure on the DBE to offer intensive training in inclusive education during the continuous professional development programmes for teachers. One other approach would be to collaborate with institutions of higher learning in support of teachers and to encourage teachers to register for short learning programmes in inclusive education and other learner support-related programmes.

The DBE should also strengthen structures such as the SBSTs, so that every teacher will be familiar with the learning support processes. Specifically, the DBE should monitor and support these processes. One such example would be to ensure that those who coordinate these structures are appointed permanently and not voluntarily. This would improve accountability and ensure that learners will be supported. Additionally, structures and systematic collaboration with stakeholders such as social workers, health practitioners and educational psychologists should be used to facilitate learning support.
